# A single reinforcement learning model to unify habit formation and Pavlovian-instrumental interaction

**DOI:** 10.1038/s41598-026-55166-9

**Published:** 2026-06-08

**Authors:** Kengo Okitsu, Yutaka Sakai

**Affiliations:** 1 Graduate School of Brain Sciences, Tamagawa University, Machida, Japan; 2 Brain Science Institute, Tamagawa University, Machida, Japan

**Keywords:** Learning algorithms, Classical conditioning, Operant learning

## Abstract

The conditioning paradigm has observed learning behaviors in animals. The theory which explains behaviors assumes that a brain appropriately combine two reinforcement learning systems, consisting of model-based and model-free system. There are two ways to combine systems: hybrid system, in which two systems work independently and are combined, and single system, in which two systems work cooperatively. While the former is widely discussed, the latter has not been sufficiently investigated in terms of its capabilities. Here, we propose a single system that integrates model-based and model-free systems to include the roles of Pavlovian conditioning. Our simulation results show that the proposed model can explain habit formation, Pavlovian conditioning, and Pavlovian-to-instrumental transfer in a unified manner. This suggests the possibility of reproducing various phenomena in a single system.

## Introduction

Animals can learn appropriate behavior to acquire better outcomes through trial-and-error experience. This learning behavior has been experimentally examined in conditioning procedures and mathematically formulated within reinforcement learning (RL) framework^1^.

It is now widely accepted that two types of RL systems underlie animal learning behavior^2^. One type is model-based RL, which learns environment models to compute the optimal behavioral policy. This type is regarded as a mechanism for acquiring goal-directed behavior. The other type is model-free RL, which directly updates the behavioral policy based on experience. It has been regarded as a mechanism to acquire habitual behavior. Since animal behavior exhibits both model-based and model-free characteristics, an extensive literature suggests that brain uses hybrid RL system. In the hybrid RL system, the model-based and model-free parts work independently, and an action is selected at each step on a merged policy (e.g. weighted average of action values^3–5^). Hence, the hybrid system needs an integration mechanism to control the weight to merge policies. This raises a question what is the optimal control of the weight.

There is another approach to understand the total integrated system: first to seek a single unified system to reproduce both model-based and model-free characteristics, and next to ground its constituent processes on neural implementation in the brain. In this approach, it is important to seek a single computational framework that accounts for diverse behaviors.

Although single RL system approaches have not yet been examined as extensively as hybrid approaches, several candidate have been proposed. One representative example is Successor Representation (SR), which estimates long-term state correlations to compute behavioral policy^6^. Since SR does not infer the state-transition rule of environment, SR does not belong to model-based RL. SR, however, separates outcome estimation from state relationships such as model-based RL, differently from standard model-free RL. This characteristics enable SR to reproduce properties of goal-directed behavior such as outcome revaluation^6,7^. Thus, SR combines elements of both model-free and model-based characteristics and is considered an intermediate RL system.

Another form of intermediate single RL system is the ‘plan-until-habit’ framework^8,9^. This framework computes the optimal behavioral policy by combining model-based forward search and model-free outcome estimation. The model-based forward search is conducted up to a finite depth, with the remaining components replaced with long-term outcome estimated in the model-free manner. The both parts are necessary to maximize the outcome in a single system. Thus, it is not a hybrid but a single intermediate system possessing both model-based and model-free characteristics.

To explore how multiple learning phenomena can be explained within such a single RL system, we reconsidered the role of Pavlovian conditioning and incorporated it into the plan-until-habit framework. Pavlovian conditioning itself does not contribute increase of outcomes. Therefore, Pavlovian conditioning should play a role in a part of outcome maximization in the instrumental conditioning. In model-free RL, the role of Pavlovian conditioning is considered as the long-term outcome prediction necessary to update behavioral policy by using temporal difference (TD) learning^1,10,11^. In contrast, model-based RL infers the state-transition rule to predict the next stimuli, which includes the next outcome prediction. Namely, Pavlovian conditioning is considered to reflect a part of the whole stimuli prediction. Both roles of ‘outcome prediction’ exist in the single system of plan-until-habit framework^8,9^, as mentioned above. Thus, we modified the plan-until-habit framework to incorporate a standard model of Pavlovian conditioning: Rescorla-Wagner model^12^.

The plan-until-habit framework will be practical in a real world including infinite possibilities. If the state is discrete and finite, model-based RLs generally exhibit the best performance by backward iterations of the optimal Bellman equation. It needs, however, iterations of estimations for all possible states. Therefore, required computational resources diverge as increase of possible states. The backward solution is not practical in a real world. In contrast, the possible states are restricted in forward search from the given state. The long-term outcome can be estimated online for the given state in the model-free manner. The combination of finite-depth forward search and model-free outcome estimation do not require massive computation. Therefore, it can be a practical solution in a real world.

Our model is a single system for the sake of outcome maximization in the instrumental conditioning by incorporating the role of Pavlovian conditioning. Hence, it is expected to reproduce both behaviors in the instrumental and Pavlovian conditioning. Since both model-free and model-based characteristics coexist within this single system, the habit formation from goal-directed to habitual behaviors might be reproduced through over learning. Moreover, Pavlovian-to-instrumental transfer (PIT)^13,14^, a well-known interaction effect between Pavlovian and instrumental learning, can be reproduced. In this paper, we tested whether our model reproduces behaviors in the habit formation, Pavlovian conditioning and PIT paradigm.

## Results

### Model

In the real world, animals choose a behavioral action for given environment. In this paper, we featured behavioral choice among discrete options, rather than precise motor controls or other continuous outputs. Because the timings to require choices might be restricted, we also supposed discrete time steps of choice timings.

The choice would depend on the current state consisting of various features of environment, for examples, strengths of sensory stimuli, distances to important places, and so on. The state representation for action choice should be factorized into multiple features to give continuous values. Hence, we supposed the current state at time *t* to be represented as a feature-pattern column vector $\boldsymbol{s}_t$. The vector elements represents the respective amounts of features.

Outcome (i.e. reward and punishment) $r_t$ is obtained as a result of the chosen action $a_t$ in state $\boldsymbol{s}_t$. We supposed state features directly associated with outcomes (e.g., water, food pellets, etc.), and consuming actions (e.g., licking, eating, etc.). The actual outcome can be obtained after the consuming actions. Therefore, the consumed outcome should be a linear summation of feature pattern $\boldsymbol{s}_t$ depending on the consuming action. Behavioral costs of actions can also be expressed in the same form. Thus, we supposed that the outcome $r_t$ is determined by $\boldsymbol{s}_t$ and $a_t$,

1 $$\begin{aligned} r_t= & \boldsymbol{u}_{a_t}^\textrm{T} \boldsymbol{s}_t, \end{aligned}$$

where $\boldsymbol{u}_{a}^\textrm{T}$ is a fixed action-dependent row vector (transposed from column vector) to determine the outcome.

In reinforcement learning theory, the optimal choice $a^*$ in state $\boldsymbol{s}'$ on Markov decision process is the action associated with the maximum action values^15^,

$$\begin{aligned} a^* = \arg \max _{a'} Q(\boldsymbol{s}',a')\equiv \arg \max _{a'} E\left[ \sum _{\tau =0}^\infty \gamma ^\tau r_{t+\tau }\Big |\boldsymbol{s}_t=\boldsymbol{s}',a_t=a'\right] . \end{aligned}$$

Therefore, the estimation of action values $\{Q(\boldsymbol{s}',a')\}$ is critical to optimize behavior. However, the action values are functions of continuous vector $\boldsymbol{s}'$, and hence the precise estimation is generally difficult. To estimate the continuous action values, we adopted a linear approximation and a combination of the model-based forward search and the model-free outcome estimation. For the learning of linear approximations, we extended Rescorla-Wagner model proposed for Pavlovian conditioning. Rescorla-Wagner model utilizes a linear regression to predict outcomes from binary stimulus patterns. This can be easily extend to continuous patterns of states. Hence, we adopted Rescorla-Wagner-type linear regressions for state-transition models and model-free outcome estimations. Namely, we used Rescorla-Wagner-type models for both of the model-based search and model-free estimation.

We assumed the model-free outcome estimation based on state values estimated by a linear regression

$$\begin{aligned} V(\boldsymbol{s}')\equiv E\left[ \sum _{\tau =0}^\infty \gamma ^\tau r_{t+\tau }\Big |\boldsymbol{s}_t=\boldsymbol{s}'\right] \approx \boldsymbol{v}^\textrm{T} \boldsymbol{s}', \end{aligned}$$

which can be estimated by the temporal difference learning^1^,

$$\begin{aligned} \boldsymbol{v} \leftarrow \boldsymbol{v} + \alpha _v (r_t + \gamma \boldsymbol{v}\cdot \boldsymbol{s}_{t+1} -\boldsymbol{v}\cdot \boldsymbol{s}_{t} ) \boldsymbol{s}_t. \end{aligned}$$

The standard model-free learning utilizes the state value one-step after the action,

$$\begin{aligned} Q(\boldsymbol{s}',a') = E[r_t + \gamma \boldsymbol{v}\cdot \boldsymbol{s}_{t+1} | \boldsymbol{s}_t=\boldsymbol{s}',a_t=a']. \end{aligned}$$

Instead, our model utilizes the state value after several steps *D*,

2 $$\begin{aligned} Q(\boldsymbol{s}',a')= & E\left[ \sum _{\tau =0}^{D} \gamma ^{\tau } r_{t+\tau } + \gamma ^{D+1} \boldsymbol{v}\cdot \boldsymbol{s}_{t+D+1}\Big | \boldsymbol{s}_t=\boldsymbol{s}',a_t=a'\right] , \end{aligned}$$

and the first several terms are estimated through the model-based forward search.

We assumed the model-based forward search based on one-step prediction of the next state pattern depending on the chosen action. We expanded RW model to incorporate action dependence such that each amount of the next state after the chosen action $a_t$ be predicted from the current state $\boldsymbol{s}_t$ with a linear regression by action-dependent and -independent coefficients, $(\boldsymbol{w}^{0}+\boldsymbol{w}^{a_t})^\textrm{T} \boldsymbol{s}_t$ (see Methods). The expectation value of the next state vector is described with a action-dependent matrix $W_{a}$ in which the action-dependent and -independent coefficients are merged,

3 $$\begin{aligned} E[\boldsymbol{s}_{t+1}|\boldsymbol{s}_t,a_t]\approx & W_{a_t}\boldsymbol{s}_t. \end{aligned}$$

**Fig. 1:**
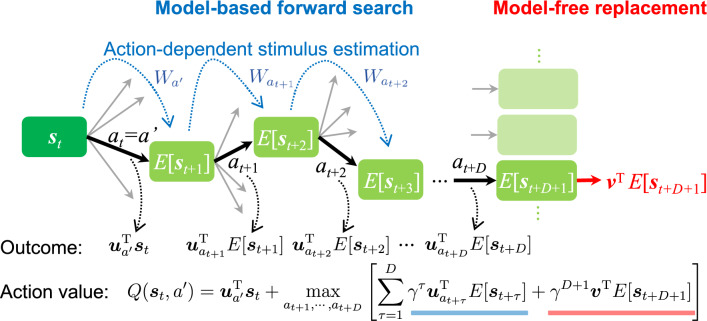
Scheme of model-based forward search and model-free value replacement to estimate the action values for a given state $\boldsymbol{s}_t$.

By using the state expectation as the input of the next state expectation, the expectations can be chained forward on a certain assumption of an action sequence $A_{t+1}^{t+\tau -1} \equiv \{a_{t+1}, \cdots , a_{t+\tau -1}\}$ (Fig. 1),

$$\begin{aligned} E[\boldsymbol{s}_{t+\tau }|\boldsymbol{s}_t,a_t,A_{t+1}^{t+\tau -1}]\approx & W_{a_{t+\tau -1}}\cdots W_{a_{t+1}}W_{a_t}\boldsymbol{s}_t. \end{aligned}$$

The outcome expectation is estimated on a certain action sequence by using equation (1),

$$\begin{aligned} E[r_{t+\tau }|\boldsymbol{s}_t,a_t,A_{t+1}^{t+\tau }] \approx \boldsymbol{u}_{a_{t+\tau }}^\textrm{T} W_{a_{t+\tau -1}}\cdots W_{a_{t+1}}W_{a_t}\boldsymbol{s}_t. \end{aligned}$$

Thus, the first several terms of the action value in equation (2) can be estimated through the chain of state expectations on a certain action sequence,

4 $$\begin{aligned} Q(\boldsymbol{s}_t,a_t,A_{t+1}^{t+D})\equiv & \sum _{\tau =0}^{D} \gamma ^{\tau } \boldsymbol{u}_{a_{t+\tau }}^\textrm{T} \prod _{t'=t}^{t+\tau -1} W_{a_{t'}} \boldsymbol{s}_t + \gamma ^{D+1} \boldsymbol{v}^\textrm{T} \prod _{t'=t}^{t+D} W_{a_{t'}} \boldsymbol{s}_t. \end{aligned}$$

All possible action sequences from the next to the depth *D*, $A_{t+1}^{t+D}$, are searched, and the action value are estimated as the maximum,

5 $$\begin{aligned} Q(\boldsymbol{s}_t,a_t)\approx & \max _{A_{t+1}^{t+D}} Q(\boldsymbol{s}_t,a_t,A_{t+1}^{t+D}). \end{aligned}$$

The action-value estimation by forward search and state-value replacement is performed every step, and the state-dependent choice is determined by soft-max choice probabilities,

6 $$\begin{aligned} P(a_t=a'|\boldsymbol{s}_t)= & \frac{e^{\beta Q(\boldsymbol{s}_t,a')}}{\sum _{a \in \mathcal {A}(\boldsymbol{s}_t)} e^{\beta Q(\boldsymbol{s}_t,a)}}, \end{aligned}$$

where $\mathcal{A}(\boldsymbol{s}_t)$ is the set of available actions in state $\boldsymbol{s}_t$.

### Demonstration of conditioning behavior

**Fig. 2:**
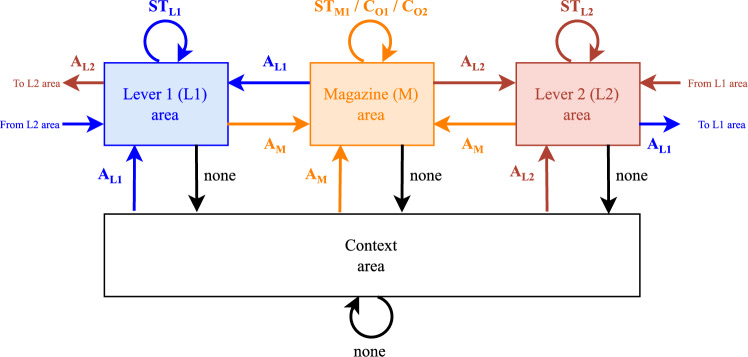
Schematic of transitions between action areas. Blue, orange, and red lines indicate moving actions related to L1, M, and L2, respectively. “none” indicates that the agent selects the context action (i.e., moves to the context area).

To represent an environment of an operant chamber, we introduced state features to represent “action area” (Fig. 2). Action area constrains available actions. Press of a certain lever is available only in the lever area (L1 or L2). Outcome consuming is available only in the outcome magazine area (M). We supposed “context area” (N) irrelevant to the conditioning in a given experimental context. Inner-areal stay $\textrm{ST}_\textrm{X}$ and inter-areal movements $\textrm{A}_\textrm{X}$ are also available actions.

The action area is a state-like concept that indicates that a specific Pavlovian or instrumental action was selected one time step ago. In our simulation, we assume that the agent transitions deterministically among the action areas. There are two types of actions associated with an action area: approaching action, which moves the agent from one action area to another, and staying action, which keeps the agent in previous action area. An approaching action and staying action are collectively called “moving action”. When the agent selects a moving action, it moves to the corresponding action area. For each action area, the actions that could be selected by the agent are different. Besides, the agent observes a stimulus that indicates which action area it is in. We treated moving to the context area (N) as “context action” (none) which represents default actions irrelevant to the conditioning.

To represent Pavlovian and instrumental conditioning, we set up three action areas in addition to the context area, *\mathrm L1* as Lever 1, *\mathrm L2* as Lever 2 and *\mathrm M* as Magazine. The stimulus set for this simulation is as follows:

$$\begin{aligned} \mathcal{S}=\{\text{ N },\textrm{S1, S2, O1, O2, L1, L2, M}\}, \end{aligned}$$

where L1, L2, and M are the stimulus that indicates action area. The action set is as follows:

$$\begin{aligned} \mathcal{A}=\{\text{ none },\mathrm{A_{L1}, A_{L2}, A_{M}, ST_{L1}, ST_{L2}, ST_{M}, C_{O1},C_{O2}}\}, \end{aligned}$$

where none is the context action, $\mathrm {A_{L1}}$, $\mathrm {A_{L2}}$, and $\mathrm {A_{M}}$ are approaching action, $\mathrm {ST_{L1}}$, $\mathrm {ST_{L2}}$, and $\mathrm {ST_{M}}$ are staying action, and $\mathrm {C_{O1}}$ and $\mathrm {C_{O2}}$ are consuming action. Figure 2 illustrates the action area transition. Here we describe the area transition in instrumental conditioning as an example. The agent starts from a context area and can select the approaching action ($\mathrm{A_{L1}, A_{L2}, A_{M}}$) corresponding to each action area.

Throughout the simulations, we set each interval of time steps as 1 s. Table 1 shows the protocol design of simulations. For Pavlovian, instrumental and extinction phase, the training schedule per session is the same for all protocols. In a Pavlovian session, 2-min CSs is presented 6 times and during each 2-min presentation, four food pellets are delivered according to a random time (RT) 30 schedule. In 30 minutes of an instrumental session, presses on the active lever are rewarded according to a random interval (RI) 30 schedule. In 20 minutes of an extinction session, outcomes are not delivered for any instrumental actions. For all protocols, the parameter settings are the same and 10 samples of simulation were run for each condition. See Environment settings for demonstrations for the details of the simulations.

#### Habit formation

**Fig. 3:**
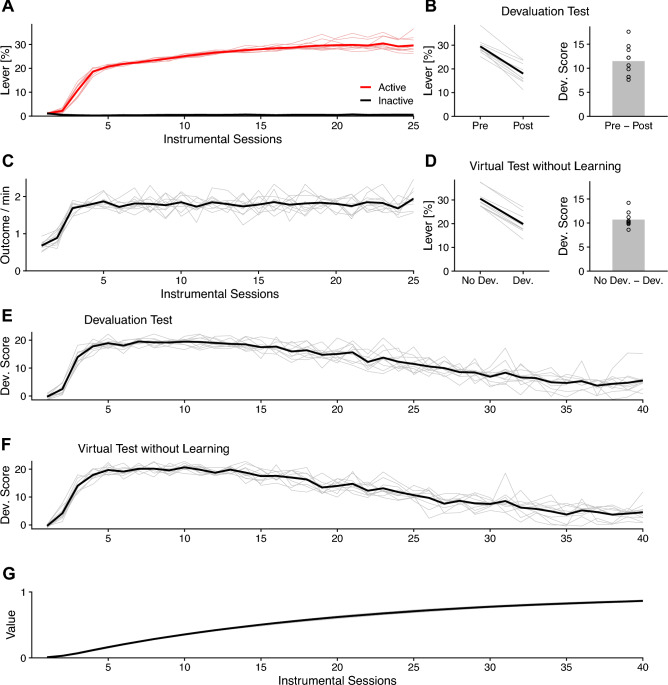
Habit formation simulation results. For all line graphs, light-colored line indicates the results of each sample of simulations. (**A**) Average rates of instrumental moving actions for active and inactive levers across sessions of instrumental training on 25 session condition. (**B**) Left panel shows action rate for active lever during pre and post devaluation test on 25 session condition. In right panel, bar plot shows average devaluation score on 25 session condition, and round points shows devaluation score for each simulation sample. (**C**) Average reinforcement rates across sessions of instrumental training on 25 session condition. (**D**) Left panel shows active lever rate for No Dev. and Dev. conditions of virtual devaluation test on 25 session condition. In right panel, bar plot shows average devaluation score of virtual devaluation test on 25 session condition, and round points shows devaluation score for each simulation sample. (**E** and **F**) The evolution of the devaluation score across 1 to 40 session conditions in the devaluation test or virtual devaluation test. (**G**) Average value expectation of outcome cross sessions of instrumental training on 40 session condition.

To investigate the degree of devaluation as a function of the number of sessions of instrumental training, we conducted the simulations of habit formation protocols. In this protocol, the 5-min devaluation test was conducted after 1 to 40 sessions of instrumental training followed by a devaluation. In the devaluation phase, the outcome value for combination of outcome *\mathrm O1* and consuming action $\mathrm {C_{O1}}$ was set to *-1* from 1. After devaluation test, devaluation score was calculated by subtracting action rate during last 5 minutes of instrumental training (Pre devaluation) from action rate during devaluation test (Post devaluation). Figure 3A-C shows an example of a devaluation score calculation for the 25 session condition. It is clear that instrumental conditioning is established for active lever (Fig. 3A, C). As Fig. 3B indicates, under 25 session condition devaluation effect has occurred.

To test whether this effect occurs immediately after devaluation, we conducted virtual devaluation test. In the virtual devaluation test, the simulation protocol is almost the same as devaluation test above. The difference is that learning all parameters were stopped after instrumental training, and two types of devaluation test were conducted. No Devaluation (No Dev.) condition is the devaluation test without outcome devaluation, and Devaluation (Dev.) condition is the devaluation test with outcome devaluation. In the case of virtual devaluation test, devaluation score was calculated by subtracting action rate during devaluation test under Dev. condition from action rate during devaluation test under No Dev. condition. It is clear that under 25 session condition devaluation effect occurs immediately after devaluation (Fig. 3D).

Figure 3E and F show that devaluation scores decrease as the number of instrumental training sessions increases, in both the devaluation and virtual devaluation tests. These results suggest that a training-dependent shift in behavioral control is reproduced in the proposed single-system model. Figure 3G further shows that the model-free outcome-value expectation changes across training under the 40-session condition, implying that the reduced devaluation effect is accompanied by progressive changes in the model-free estimation.

The model-free value estimation needs the large number of training sessions, because it should predict long-term outcomes. In contrast, inference of step-by-step transitions converges in less number of sessions. Therefore, the improvement in model-based forward prediction contributes dynamics of the response rate in early training sessions, and the relative contribution of model-free estimation gradually becomes larger. It is considered that the change of the relative contribution reflects the decrease of the devaluation effect. Taken together, the present results showed that the habit formation can emerge as learning dynamics without explicit changes of learning parameters.

#### Pavlovian conditioning

**Fig. 4:**
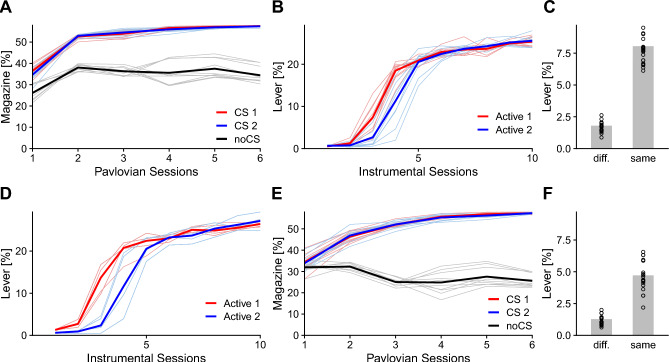
Simulation results for specific PIT with two outcomes. For all line graphs, light-colored line indicates the results of each sample of simulations. **A-C** shows the results derived from the simulation of Pavlovian training followed by instrumental training, and **D-F** shows them derived from the simulation of instrumental training followed by Pavlovian training. (**A** and **E**) Average rates of Pavlovian moving actions during CSs or no-CS presentation on 6 Pavlovian session condition. (**B** and **D**) Average rates of instrumental moving actions for active levers on 10 instrumental session condition. (**C** and **F**) bar plot shows average rates of instrumental moving actions for active levers on diff. and same condition, and round points shows action rate for each simulation sample.

To confirm whether our model reproduces Pavlovian conditioning, we simulated the model under a protocol of Pavlovian conditioning (CS1 *→* reward; CS2 *→* no; see Methods). We confirmed that the ratio of magazine approaching actions for CS1 became larger than that for CS2 (Fig. 4A).

The reason for the reproducibility of Pavlovian conditioning was due to the areal settings and the temporal discounting. The reward consumption needs two actions, magazine approaching and consuming. The beforehand approach enables early consumption. The degree of magazine approaching depends on the temporal discounting rate and the difference between the cost of approaching and staying actions for the magazine.

#### Specific PIT

To investigate whether Pavlovian-instrumental interaction occurs, we simulated a specific PIT protocol with two outcomes. In this protocol, the transfer test was conducted after Pavlovian and instrumental training followed by extinction. During the transfer test, each Pavlovian CS (S1 and S2) was presented four times in a pseudo-random order for 2-min, with a fixed 2-min inter-trial interval. One of the two instrumental actions was available, and its action rate during CS presentation was measured. From the perspective of specific transfer, we defined two action rate conditions: “same” and “diff.”, depending on whether the CS and the lever were associated with the same outcome or different outcomes. A specific transfer effect was defined as a higher action rate in the same condition than in the diff. condition.

Figure 4 shows the simulation results under two different training-order protocols. In both protocols, Pavlovian and instrumental conditioning were established, and the action rate is higher in the same condition than in the diff. condition. These patterns suggest that a specific PIT-like effect is reproduced in the proposed model across training orders.

Interaction among different protocols was caused by the linear regression forms. Weights for the outcomes O1 and O2 were common among the protocols, and affected responses through linear summations with the other terms. In the test phase, outcome predictions related to CS presentation and lever pressing are combined, and this integration increases the state-action value of CS-lever combinations linked to the same outcome. Because only one type of outcome can be consumed at each step, this additive influence remains selective with respect to outcome identity. Taken together, these properties suggest that responding is enhanced in the same condition relative to the diff. condition and imply an outcome-selective mechanism for specific PIT in the proposed model.

**Fig. 5:**
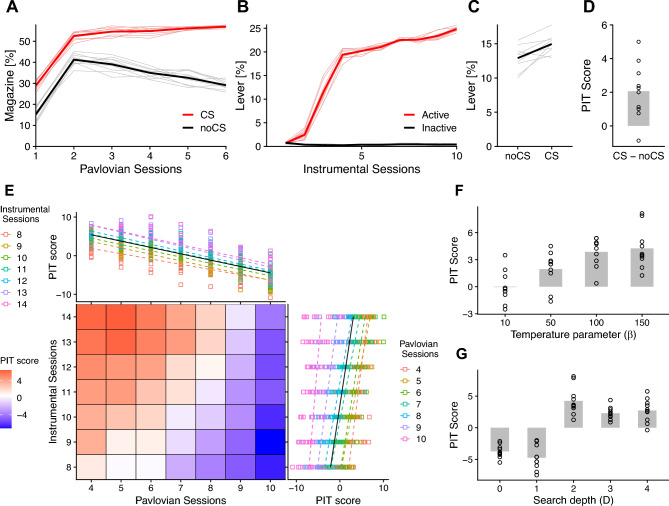
Simulation results for specific PIT with one outcome. For all line graphs, light-colored line indicates the results of each sample of simulations. **A-D** shows the results derived from the simulation of Pavlovian training followed by instrumental training. (**A**) Average rates of Pavlovian moving actions during CS or no-CS presentation on 6 Pavlovian session condition. (**B**) Average rates of instrumental moving actions for active and inactive levers across sessions of instrumental training on 10 instrumental session condition. (**C**) Average action rate for active lever during CS or no-CS presentation at PIT test. (**D**) The bar plot shows average PIT score at PIT test, and round points shows PIT score for each simulation sample. (**E**) The heat map plot shows the average PIT score for each condition. Two marginal plots show trends in PIT scores for Pavlovian or instrumental sessions. The scatter plot plots PIT scores for each simulation sample. The dashed line represents the results of linear regression when grouped by Pavlovian or instrumental session. The solid lines represent the results of linear regression for all samples. (**F**) Qualitative robustness for temperature parameter *β*. (**G**) Qualitative robustness for search depth *D*.

We also confirmed qualitative robustness for learning parameters (Fig. 5F for temperature parameter *β*, G for search depth *D*). The low *β* hided the effect of PIT because it increases random responses, whereas qualitative tendency was kept (Fig. 5F). The short depth *D<2* could not reproduce PIT (Fig. 5G). That is because forward search up to the outcomes is necessary for the summation mechanism mentioned above. We confirmed the robustness for the longer depths *D=2, 3, 4*.

#### Pavlovian and Instrumental action competition

In addition to testing whether the model reproduces PIT, we examined whether it also reproduces the training-dependent interaction reported previously, namely that PIT score decreases as Pavlovian training increases and increases as instrumental training increases in a PIT protocol in which Pavlovian training precedes instrumental training^13^. We conducted simulations of a specific PIT protocol with a single outcome, systematically varying the numbers of Pavlovian and instrumental training sessions to examine their effects on PIT scores. For each combination of Pavlovian and instrumental training sessions, we calculated PIT score as the difference between action rate during CS presentation and that during the inter-CS interval. Thus, positive PIT scores indicate CS-induced enhancement of instrumental responding, whereas negative scores indicate suppression. Figure 5C and D show an example of this calculation, and Fig. 5E summarizes PIT scores across training conditions. As shown in Fig. 5E, PIT scores tend to decrease with increasing Pavlovian training and increase with increasing instrumental training.

It is considered that CS presentation enhances both Pavlovian and instrumental action tendencies, and that the net PIT score reflects competition between these two tendencies. When Pavlovian training is stronger, CS-evoked Pavlovian action tends to be relatively dominant, reducing instrumental responding during CS and lowering PIT scores. When instrumental training is stronger, the instrumental action tendency tends to become relatively dominant, increasing responding during CS and elevating PIT scores. These relationships suggest that the observed training-dependent PIT modulation emerges from a balance shift between Pavlovian and instrumental influences within the proposed single-system model.

## Discussion

The present study explored whether multiple conditioning phenomena can be explained within a single RL system that possesses both model-based and model-free characteristics. A large body of previous work has explained such behavioral diversity using hybrid architectures, in which model-based and model-free components are computed separately and then integrated for action selection. In those frameworks, behavioral changes such as habit formation can be reproduced by adjusting explicit integration parameters that control the balance between the two components^3–5^. They leave a question how the balance should be controlled. By contrast, our model reproduces habit formation, Pavlovian conditioning, and Pavlovian-to-instrumental transfer (PIT) within a single computational system, without requiring explicit changes of such integration parameters across phenomena. More precisely, although the search depth *D* affects the balance between model-based and model-free contributions, the present results were obtained without changing explicit integration weights or other mechanism-selection parameters between simulations. This difference is important because it suggests that diverse learning phenomena may emerge through learning within a unified system, rather than through explicit changes in integration parameters that regulate the balance between separate systems. However, the relative merits of single-system and hybrid approaches remain an open question that future empirical and computational work should address. In this regard, the present model provides a complementary framework for exploring such questions.

The proposed model is based on the plan-until-habit framework^8,9^, but it is not a mere restatement of that framework. The basic architecture is shared: model-based forward search is combined with a model-free replacement term within a single decision process. However, there are two important differences. First, we introduced action-dependent linear regression of state transitions for forward searches. This allowed generalization among Pavlovian and instrumental conditions. Second, our model replaced the distant parts of the forward search with the state values that do not indicate ‘habits’ themselves, whereas the original plan-until-habit framework replaces those with ‘habits’ representations (action values). Thus, the present model preserves the basic idea of coordinated model-based and model-free computation, while introducing a distinct representational and computational scheme.

Another important contribution of the present study is the incorporation of Pavlovian conditioning into this single-system framework. The original plan-until-habit framework was not designed to reproduce Pavlovian behavior itself, because it did not explicitly include Pavlovian action structure or environmental settings such as the magazine area in an operant chamber. In the present model, we introduced action-area representations and magazine-related action constraints, and we reconsidered Pavlovian conditioning as a process relevant not only to model-free long-term outcome prediction but also to model-based stimulus prediction. This extension allowed Pavlovian learning to influence instrumental choice within the same computational framework. Therefore, the significance of the present model is not only that it reproduces instrumental phenomena in a single system, but also that it incorporates Pavlovian processes into that system and thereby accounts for interactions between Pavlovian and instrumental learning.

Within this framework, habit formation emerged because the contribution of model-free value learning increased through training, while the model-based component continued to contribute through finite-depth forward search. This provides a mechanistic explanation of how a behavioral shift from goal-directed to habitual responding can arise without changing explicit integration weights between separate systems. Likewise, specific PIT was reproduced because Pavlovian and instrumental learning jointly contributed to outcome prediction, and because the model preserved outcome identity through its action- and state-based representation. The model also reproduced competition between Pavlovian and instrumental actions, indicating that the same framework can account not only for facilitation of instrumental responding by Pavlovian cues but also for training-dependent modulation of that interaction.

The biological plausibility of the proposed framework should also be considered. Recent studies have argued that temporal-difference learning implemented directly through dopamine-like global teaching signals may not be biologically realistic in its standard form, while functionally similar learning may still be achieved through local synaptic plasticity mechanisms such as spike-timing-dependent plasticity^16,17^. From this perspective, the model-free state-value learning component of our model may be related to dopamine-dependent learning, whereas other components, including the learning of state transitions and forward prediction, may be implemented by local synaptic plasticity. Although this interpretation remains speculative, it suggests that the present framework is not necessarily inconsistent with biologically plausible learning mechanisms.

Several limitations should be noted. First, although the present model reproduces multiple phenomena within one framework, the balance between model-based and model-free influences is still affected by the search depth *D*. Thus, our claim is not that the model is free of parameters affecting this balance, but rather that the reproduced phenomena did not require explicit re-tuning of integration parameters across tasks. Second, the present simulations relied on simplified environmental settings, including action areas and magazine constraints, which were introduced to represent essential features of conditioning paradigms. Future work should test whether the same framework can explain broader classes of behavior under richer and less structured environments. It will also be important to examine experimentally whether assumptions of the model, such as outcome-selective consumption and the interaction between Pavlovian and instrumental response tendencies, are supported in animal behavior.

In sum, we proposed a single RL model that extends the plan-until-habit framework by introducing action-dependent state prediction and by incorporating the role of Pavlovian conditioning. The model differs from hybrid accounts in that diverse behavioral phenomena emerge through learning in a unified system rather than through explicit adjustment of integration parameters between separate systems. Our results suggest that a single intermediate RL system can provide a useful framework for understanding habit formation and Pavlovian-instrumental interaction in a unified manner.

## Methods

### Environment definition

We featured action choices among discrete options at discrete timings for continuous states. We formulated the model in discrete time step by a fixed interval, rather than event-based discrete timings or continuous time. We attempted to cover continuous-time features in real experiments by a sufficiently short interval of time steps. The state consists of multiple features of the environment. For example, sensory stimuli, positions, and availability of instruments can be candidates of state features. Suppose a finite set of state features, $\mathcal {S}=\{F_0,F_1, \cdots , F_{n-1} \}$, and a state vector $$\boldsymbol{s}= \begin{pmatrix} s_0&s_1&\cdots&s_{n-1} \end{pmatrix}^\textrm{T}$$ to represent respective amounts of the state features. The first feature $F_0$ is special such that always $s_0=1$. It is, so to speak, a null feature which works as a constant term in a linear regression, as described later.

Suppose a finite set of available actions, $\mathcal {A}^\textrm{all}=\{A_1, A_2, \cdots , A_{m}\}$. When the chosen action $a_t=i$ means that it is *i*-th action $A_i$. Action availability can be restricted by certain state features, $\mathcal {A}(\boldsymbol{s}) \subset \mathcal {A}^\textrm{all}$.

The outcome $r_t$ is described with a fixed action-dependent vector $\boldsymbol{u}_{a}$, $r_t=\boldsymbol{u}_{a_t}^\textrm{T}\boldsymbol{s}_t$, as in the equation (1) of the main text. The purpose of learning is to maximize the average outcome $E[r_t]$.

### Action-dependent linear regression

In this paper, we assumed the model-based forward search based on one-step prediction of the next state pattern depending on the chosen action. We expanded the Rescorla-Wagner model to incorporate action dependence because instrumental conditioning requires optimization over candidate actions. Each amount of the next state after the chosen action $a_t$ be predicted from the current state $\boldsymbol{s}_t$ with a linear regression by action-dependent and -independent coefficients,

7 $$\begin{aligned} E[(\boldsymbol{s}_{t+1})_j|\boldsymbol{s}_t,a_t]\approx & \sum _{k=0}^n \left( w^0_{jk} + w^{a_t}_{jk} \right) (\boldsymbol{s}_t)_k, \end{aligned}$$

where $(\boldsymbol{s}_t)_k$ means the *k*-th element of vector $\boldsymbol{s}_t$. The decomposition into action-independent and action-dependent coefficients can be interpreted as a baseline-plus-deviation form in linear regression. The action-independent term captures stimulus-to-next-state structure shared across actions, whereas the action-dependent term captures action-specific deviations (i.e., action-stimulus interaction effects). This decomposition allows common regularities to be shared while preserving differences among actions. The *jk*-element of the action-dependent matrix $W_a$ in equation (3) of the main text corresponds to $(W_a)_{jk}=w_{jk}^0+w_{jk}^a$. For each amount of the next state, the action-independent and -dependent coefficients are updated at every step according to the manner of Rescorla-Wagner model,

8 $$\begin{aligned} w_{jk}^0 \leftarrow w_{jk}^0 + \alpha _{w} \left[ s_j(t+1) - \left( W_{a_t} \boldsymbol{s}_t\right) _j \right] (\boldsymbol{s}_t)_k, \nonumber \\ w_{jk}^{a_t} \leftarrow w_{jk}^{a_t} + \alpha _{w} \left[ s_j(t+1) - \left( W_{a_t} \boldsymbol{s}_t\right) _j \right] (\boldsymbol{s}_t)_k. \end{aligned}$$

**Table 1:** Protocols of simulation.

**Habit formation**			
Instrumental (1–40 session)	Devaluation	Test	
L1-O1	O1	L1	
L2-			

**Specific PIT (tow outcomes)**			
Pavlovian *→* Instrumental			
Pavlovian (6 session)	Instrumental (10 session)	Extinction	Test
S1-O1	L1-O1	L1-	S1:L1/L2
S2-O2	L2-O2	L2-	S2:L1/L2

Instrumental *→* Pavlovian			
Instrumental (10 session)	Pavlovian (6 session)	Extinction	Test
L1-O1	S1-O1	L1-	S1:L1/L2
L2-O2	S2-O2	L2-	S2:L1/L2

**Specific PIT (single outcome)**			
Pavlovian (4–10 session)	Instrumental (8–14 session)	Extinction	Test
S1-O1	L1-O1	L1-	S1:L1/L2
	L2-	L2-	

### Environment settings for demonstrations

To represent the environment of an operant chamber for Pavlovian or instrumental conditionings, we introduced state features to represent “action area”, in addition to sensory stimuli for conditionings. Action area constrains available actions. Pressing of a certain lever is available only in the lever area. Outcome consuming is available only in the outcome magazine area. Inner-areal stay and inter-areal movements are also available actions. In addition, we supposed “context area” (N) irrelevant to the conditioning in a given experimental context.

The action area is a state-like concept that indicates that a specific Pavlovian or instrumental action was selected one time step ago. Throughout the simulations, we set each interval of time steps as 1 s. In our simulation, we assume that the agent transitions deterministically among the action areas. There are two types of actions associated with an action area: approaching action, which moves the agent from one action area to another, and staying action, which keeps the agent in previous action area. An approaching action and staying action are collectively called “moving action”. When the agent selects a moving action, it moves to the corresponding action area. For each action area, the actions that could be selected by the agent are different. Besides, the agent observes a stimulus that indicates which action area it is in. We treated moving to the context area (N) as “context action” (*a=0*) which represents default actions irrelevant to the conditioning. The state of the context area is represented by the only constant term, $\boldsymbol{s}=(1,0,\cdots )$. In the same way, the context action updates only the constant term $w_{jk}^{0}$ in eq. (8).

To represent Pavlovian and instrumental conditioning, we set up three action areas in addition to the context area, *\mathrm L1* as Lever 1, *\mathrm L2* as Lever 2 and *\mathrm M* as Magazine. The stimulus set for this simulation is as follows:

$$\begin{aligned} \mathcal{S}=\{\text{ N },\textrm{S1, S2, O1, O2, L1, L2, M}\}, \end{aligned}$$

where L1, L2, and M are the stimulus that indicates action area. The action set is as follows:

$$\begin{aligned} \mathcal{A}=\{\text{ none },\mathrm{A_{L1}, A_{L2}, A_{M}, ST_{L1}, ST_{L2}, ST_{M}, C_{O1},C_{O2}}\}, \end{aligned}$$

where $\mathrm {A_{L1}}$, $\mathrm {A_{L2}}$, and $\mathrm {A_{M}}$ are approaching action, $\mathrm {ST_{L1}}$, $\mathrm {ST_{L2}}$, and $\mathrm {ST_{M}}$ are staying action, and $\mathrm {C_{O1}}$ and $\mathrm {C_{O2}}$ are consuming action. Figure 2 illustrates the action area transition. Here we describe the area transition in instrumental conditioning as an example. The agent starts from a context area and can select the approaching action ($\mathrm{A_{L1}, A_{L2}, A_{M}}$) corresponding to each action area. The agent transition to L1 area if approaching action L1 is selected. In the L1 area, the agent observes a stimulus that indicates L1 area, and can choose either approaching action to move to another area or staying action $\mathrm{ST_{L1}}$ to stay in the L1 area. If outcome O1 is presented to the Magazine, the agent selects approaching action $\mathrm{A_{M}}$ to the Magazine (M) area. In the M area, the agent can choose either staying action $\mathrm{ST_{M}}$ to stay in the M area or consuming action $\mathrm{C_{O1}}$ of the presented outcome. If consuming action is selected, the agent can obtain presented outcome and stays in M area.

The following describes the parameter settings employed in the demonstrations presented in this study. First, the parameter settings associated with outcome are described below. The amount of state features was fixed at 1 when presented in the simulation, although it is represented as a continuous value in the model. The cost for inter-areal movement was set to *-0.025*, and the cost for staying within the same area (inner-areal stay) was set to half of that value, at *-0.0125*. The cost for all other actions was set to 0. Consuming actions for positive outcomes were assumed to yield an outcome value of 1. Action costs were introduced to reflect effort/time burden in behavior. Their values were tuned to match plausible action frequencies; therefore, while exact values are not unique, the relative balance between moving and staying costs is important for realistic action rates.

Next, we describe the parameter settings related to the agent’s prediction and learning. The forward search depth *D* is fixed at *D=2* to include the shortest path of the action sequence up to consuming in instrumental conditioning (i.e. $\mathrm {A_{L1} \rightarrow A_{M} \rightarrow C_{O1}}$). When a moving action for an area is selected, it is assumed that at least one instrumental or Pavlovian action (i.e. lever press or magazine visit) for that area is performed, and the rate of a moving action per minute is used as an indicator of the rate of actions for that area. The discount parameter *γ*, which determines temporal weighting of future outcomes, was fixed at 0.9 in all simulations. The temperature parameter *β* used in the soft-max function for computing action selection probabilities was set to 150. The action-independent and action-dependent coefficients used in the forward search were both initialized to 0, with learning rates set to $\alpha _{w}=0.0004$ and $\alpha _{v}=0.001$, respectively. These values were selected so that learning trajectories could be observed and approached stable behavior within the predefined number of training sessions. Because effective value updates are less frequent than transition-feature updates under the present schedules, $\alpha _{v}$ was set slightly larger than $\alpha _{w}$ to balance learning progress. As an exception, the action-independent coefficient from a given positive outcome (e.g., water, food pellets, etc.) to the same positive outcome was set to 1. This reflects the assumption that positive outcomes are already well learned through prior exposure.

## Data Availability

The code used to generate the simulation results in this study is publicly available at GitHub. No external datasets were used.
